# Serbian Version of the eHealth Literacy Questionnaire (eHLQ): Translation, Cultural Adaptation, and Validation Study Among Primary Health Care Users

**DOI:** 10.2196/57963

**Published:** 2024-05-09

**Authors:** Branko Vujkovic, Voin Brkovic, Ana Pajičić, Vedrana Pavlovic, Dejana Stanisavljevic, Dušanka Krajnović, Aleksandra Jovic Vranes

**Affiliations:** 1 Institute of Public Health of Sabac Sabac; 2 Medical Faculty University of Belgrade Belgrade; 3 Faculty of Pharmacy University of Belgrade Belgrade

**Keywords:** eHealth, digital health, eHLQ, eHealth Literacy Questionnaire, digital health literacy, primary healthcare, Serbia, questionnaire, technology, communication

## Abstract

**Background:**

As digital health services are increasingly developing and becoming more interactive in Serbia, a comprehensive instrument for measuring eHealth literacy (EHL) is needed.

**Objective:**

This study aimed to translate, culturally adapt, and investigate the psychometric properties of the Serbian version of the eHealth Literacy Questionnaire (eHLQ); to evaluate EHL in the population of primary health care (PHC) users in Serbia; and to explore factors associated with their EHL.

**Methods:**

The validation study was conducted in 8 PHC centers in the territory of the Mačva district in Western Serbia. A stratified sampling method was used to obtain a representative sample. The Translation Integrity Procedure was followed to adapt the questionnaire to the Serbian language. The psychometric properties of the Serbian version of the eHLQ were analyzed through the examination of factorial structure, internal consistency, and test-retest reliability. Descriptive statistics were calculated to determine participant characteristics. Differences between groups were tested by the 2-tailed Students *t* test and ANOVA. Univariable and multivariable linear regression analyses were used to determine factors related to EHL.

**Results:**

A total of 475 PHC users were enrolled. The mean age was 51.0 (SD 17.3; range 19-94) years, and most participants were female (328/475, 69.1%). Confirmatory factor analysis validated the 7-factor structure of the questionnaire. Values for incremental fit index (0.96) and comparative fit index (0.95) were above the cutoff of ≥0.95. The root mean square error of approximation value of 0.05 was below the suggested value of ≤0.06. Cronbach α of the entire scale was 0.95, indicating excellent scale reliability, with Cronbach α ranging from 0.81 to 0.90 for domains. The intraclass correlation coefficient ranged from 0.63 to 0.82, indicating moderate to good test-retest reliability. The highest EHL mean scores were obtained for the *understanding of health concepts and language* (mean 2.86, SD 0.32) and *feel safe and in control* (mean 2.89, SD 0.33) domains. Statistically significant differences (all *P*<.05) for all 7 eHLQ scores were observed for age, education, perceived material status, perceived health status, searching for health information on the internet, and occupation (except domain 4). In multivariable regression models, searching for health information on the internet and being aged younger than 65 years were associated with higher values of all domain scores except the domain *feel safe and in control* for variable age.

**Conclusions:**

This study demonstrates that the Serbian version of the eHLQ can be a useful tool in the measurement of EHL and in the planning of digital health interventions at the population and individual level due to its strong psychometric properties in the Serbian context.

## Introduction

### Background

The increasing digitization of the health system requires the users of health services to have sufficient competence in the use of digital health (DH) technologies. The use of DH technologies for health, also called eHealth, has brought a revolution in the way we diagnose and treat patients and take care of health. eHealth is also defined as “the use of information and communication technologies in support of health and health-related fields” [1].

eHealth is often presented as a possible solution for numerous challenges in the health system, including a lack of health personnel, an aging population, and the comorbidities of several chronic diseases [2].

Digital technologies can strengthen health systems, improve health financing and public health, and also increase the availability of health services to vulnerable groups. eHealth and the data obtained in this way are particularly useful for the prevention and control of chronic noncommunicable diseases and care for the young, as well as for older adults and for preparing a better response to future emergencies and health risks caused by climate change [3]. Digital technologies can provide a new tool for educating patients and improving their health literacy [4]. Instead of being a passive participant in health care, eHealth solutions allow individuals to be active participants [5].

It is estimated that about half of all patients search for information about their health problems on the internet before seeking professional advice [6]. It is believed that 6 out of 10 Europeans use the internet to solve health concerns, and 9 out of 10 trust the information they find [7]. This information can be wrong or incomplete, and if not critically evaluated, can lead to the adoption of behaviors that can potentially harm our health [8].

Today, digital spaces have become a central environment for communication and engagement, learning, and work, as well as for disease prevention and health promotion. This has led to the development of a new dimension of health literacy: the emergence of eHealth literacy (EHL). For the adequate implementation of eHealth services, the users of these services must be eHealth literate [9].

EHL can be defined as “the ability to search, find, understand, and evaluate health information from electronic sources and apply the acquired knowledge to approaching or solving health problems” [10]. Good EHL is directly related to improving health outcomes, reducing health care costs, increasing users’ motivation to seek health information, having better knowledge about chronic diseases, adopting preventive health behaviors, and having better self-perception and care for one’s health [6-8].

As a superdeterminant of health, EHL has added significant complexity to the way users of health system services, health workers, and digital technologies interact. Health portals and telehealth systems have enabled service users to communicate remotely with service providers; cloud-based electronic health records have enabled patients to manage diagnostic data with clinicians; and wearable devices and apps have enabled users to self-manage their conditions. The increased complexity of interacting in the digital world requires additional skills and competencies from people using eHealth. However, with the increased complexity of the DH landscape, scholars have called for a more comprehensive view and have included elements related to users’ cognitive skills, communication elements, social and cultural context, or system-level attributes [11-13]. Some researchers talk about DH literacy as an evolved concept of EHL and its impact on health, but if we take into account the existence of analog computing, EHL is definitely a more correct term, and as a relatively new area, it needs further research [14].

The COVID-19 pandemic has made it clear how important it is to have access to digital platforms. Digital technologies, such as mobile phones, have made it possible to quickly trace contacts, check symptoms, seek advice, obtain necessary information, and engage in public communication and education. Access to digital spaces is particularly important when mobility is limited or when people live in rural or remote areas. Compared to traditional communication strategies, digital spaces support “accessibility and expansion of access to health information to diverse population groups, regardless of age, education, race or ethnicity, and location” and may encourage further development of health literacy [15,16]. On the other hand, a meta-analysis by Estrela et al [17] showed that EHL is significantly related to sociodemographic, economic, and cultural factors.

The rapid transition to online health services and digital communication with health professionals due to the COVID-19 pandemic has accelerated the level of digital exclusion in certain population groups, such as those with low levels of electronic literacy or insufficient access to digital devices [18].

The benefits of digitization in health care may be unused due to unequal opportunities to use digital resources [19]. Recent studies have shown that EHL interventions have a positive effect on the health and health care of older adults, and therefore it is important to provide support and guidance to older adults to narrow the aging technology gap [20,21].

### Previous Work

Regardless of the population of interest, the need for reliable internet navigation is particularly important for health issues where the consequences of using poor quality, misleading, or false information are high [22]. By providing tools and resources to evaluate online health information and critically evaluate eHealth resources, we offer an opportunity to protect consumers from harm while empowering them [12,13]. To provide relevant tools to help users navigate eHealth, an understanding of what health care users have at the outset, or their EHL, is required.

EHL is generally lower among older adults with chronic noncommunicable diseases and is characterized by reluctance to change their usual health care routines and concerns that mobile health apps will replace doctors’ visits. On the other hand, a study by Kouri et al [22] showed that mobile health innovations have the potential to help the older adult population manage chronic diseases more effectively.

In the era of digitization of health systems, EHL is a significant predictor of an individual’s health condition. As a determinant of health, EHL is important in the analysis of the health system of each country. Previous research has shown that a satisfactory level of EHL is needed for citizens to actively participate in making correct health decisions and participating in health care [23].

Study in the field of EHL has attracted the attention of a significant number of researchers, both in the field of health care and in other areas of public life. A low level of EHL exposes both the individual and an entire society to loss (health, economic, and social loss). Strategies to strengthen EHL should be developed as part of lifelong learning skills, and health care professionals should embrace improving EHL as part of regular patient care activities [24].

To develop and implement strategies to strengthen EHL, an adequate instrument for measuring EHL is needed, which also allows evaluation of the effects of implementing those strategies on increasing EHL at the individual and population levels [25]. The Serbian government prioritizes promoting health care and citizen health through digitization of services, as stated in the “digitalization program in the health care system of the Republic of Serbia” [26]. This includes connecting facilities with advanced software for secure data exchange and enabling consumers to use information and communication technologies for health support, but it is also necessary to empower users of health services to “use information and communication technologies in support of health and health-related fields” [1,26]. As eHealth services are increasingly developing and becoming more interactive in Serbia, and everywhere in the world, a comprehensive instrument for measuring EHL is necessary [27].

The eHealth Literacy Questionnaire (eHLQ) is currently licensed for use in more than 12 countries, and its ongoing translations and cultural adaptations indicate that the instrument is robust across various contexts [28]. However, the instrument has not yet been translated into Serbian.

### Goal of This Study

This study aimed to translate, culturally adapt, and investigate the psychometric properties of a Serbian version of the eHLQ; to evaluate EHL in the population of primary health care (PHC) users in Serbia; and to explore factors associated with EHL.

## Methods

### Study Design

This was a cross-sectional study conducted from April 1 to April 30, 2023, in 8 state-owned PHC centers in the territory of the Mačva district in Western Serbia. Both qualitative and quantitative research methods were used in this study.

### Data Collection

The participants of this study were PHC consumers who were recruited from PHC centers waiting rooms in 8 local municipalities of the Mačva administrative district in Western Serbia, were aged older than 19 years, and had adequate cognitive abilities to fill out the questionnaire independently. We used a stratified sampling method. In the first step, the variable for stratification was the type of settlement. By dividing into urban and rural settlements, 2 strata were obtained. In the second step, the variable for stratification was the age of the respondents, and by dividing them into young, middle-aged, and older respondents, 6 strata were obtained. The selection of respondents was carried out proportionally to the size of the stratum. The required number of respondents to examine the psychometric properties of the EHL eHLQ was 475, as defined by the questionnaire’s creator [28]. The criteria for exclusion from the research were respondents aged younger than 19 years, a health condition that prevented a respondent from filling out the questionnaire, if the Serbian language was not a respondent’s first language, illiterate persons, and refusal of participation by a respondent.

Further, 3 trained examiners delivered paper-based questionnaires to all consumers of PHC who met the criteria for inclusion in this study at the PHC centers’ waiting rooms. Participants had to fill out the questionnaire at the PHC center. Since the topic of our study included digital use and literacy competencies, we chose to consistently administer paper-based questionnaires.

The data collection consisted of administering the Serbian eHLQ along with asking general questions about owning a digital device and seeking health information on the internet.

We also collected demographic data on participants’ age, sex, education, work situation, and health status.

### Ethical Considerations

Ethical approval was granted by the Ethical Review Board at the Medical Faculty, University of Belgrade (17/IX-5) and the Ethics Committees of PHC institutions. Participation was voluntary and anonymous. All participants gave informed consent to participate in this study, and all data were anonymized. No compensation was offered to the respondents. All steps were managed according to the ethical principles described in the Helsinki Declaration [29].

### About eHLQ

The eHLQ contains 35 items on 7 scales representing the EHL framework domains: using technology to process health information, understanding health concepts and language, ability to actively engage with digital services, feeling safe and in control, being motivated to engage with digital services, access to digital services that work, and digital services that suit individual needs. Each scale consists of 4 to 6 items on a 4-point Likert scale ranging from “strongly disagree” to “strongly agree.” Scale scores range from 1 to 4, calculated on an index by averaging item scores within each scale with equal weighting. Each scale is presented separately, and no overall eHLQ score is calculated. Higher scores indicate higher abilities [28].

### Translation, Cultural Adaptation, and Pretesting

The license to translate the English version of the eHLQ to Serbian (TE2203IG) was obtained from Swinburne University, Australia. As required by the eHLQ developers, we used the Translation Integrity Procedure (TIP) to maintain equivalence between the original (English) and translated (Serbian) versions of the instrument while ensuring the linguistic and cultural appropriateness of the Serbian version. The process was further facilitated by using clear “item intent” descriptions [30,31].

One of the eHLQ’s developers (Lars Kayser) chaired the TIP process. The translation and adaptation team included 2 native Serbian forward translators (AM and RJ), 1 native English back translator (DZ), a cognitive interviewer team, and academic professionals (BV, VB, AJV, and AP), all fluent in Serbian and English. All 3 translators had excellent English and Serbian language skills. The translation and cultural adaptation process involved 3 steps (the first 2 steps were forward translation and item intent description, and the third was back translation). (1) The original English eHLQ questionnaire was translated independently into Serbian by the 2 forward translators. (2) The translators then used the item intent descriptions, which thoroughly explained the intent of each item and scale, as a guide when synthesizing their translations. During the following team discussion, the best statements for each item were chosen and combined to form the first version of the Serbian eHLQ. (3) The first version of the Serbian eHLQ was back-translated by a native English-speaking translator who had never seen the original version of the instrument. The Serbian-to-English back-translation was then compared with the English version of the eHLQ, and the items were discussed by the team to achieve consensus on the preferred version of the Serbian eHLQ. The preferred Serbian eHLQ was then tested using cognitive interviews.

Cognitive interviewing is valuable for ensuring accurate interpretations of items when translating and validating a questionnaire in another language and culture. The process of cognitive interviewing allows researchers to discover and correct items that are not interpreted as intended, thereby avoiding the future collection of inaccurate data. Cognitive interviewing does not require a large sample size, but the sample should represent demographic variety [31,32].

Cognitive interviews were conducted with 20 adults (12 female individuals) aged 27 to 63 (median 50, IQR 37-59) years, with varying educational backgrounds. The respondents were given a printed version of the questionnaire and were carefully observed while answering the items. The interviewer (BV or AP) then went through each item with the individual respondents, focusing on items the respondents appeared to find difficult. The main questions were as follows: “what were you thinking about when you were answering that item?” and “can you tell me why you selected that answer?” Participants were encouraged to elaborate on their interpretations of the items. A protocol was used for making notes during the interviews, which were also recorded, transcribed, and analyzed using a text summary [31].

Results from the cognitive interviews revealed that although most items were understood as intended, minor revisions were needed to clarify a few items and instructions. The following corrections were made throughout the Serbian eHLQ:

- The Serbian term “organizujem informacije o svom zdravlju” (organize my health information) was replaced by “upravljam informacijama o svom zdravlju” (manage information about my health) in item 25.
- The Serbian term “u sisteme zdravstvenih tehnologija” (into health technology systems) was replaced by “u digitalne zdravstvene sisteme” (into eHealth systems) in item 8.

When we reached an agreement on all formulations, the final version of the Serbian eHLQ was considered ready to be psychometrically tested.

### Statistical Analysis

Descriptive statistics were calculated to determine participant characteristics. The psychometric properties of the Serbian version of the eHLQ were analyzed through the examination of factorial structure and internal consistency (reliability). Confirmatory factor analysis (CFA) was performed to confirm the original 7D structure of the eHLQ. The absolute goodness-of-fit of the 7D model was evaluated using the *χ*^2^ test (*P* values that are <.05 signify that a model may be a bad fit for the data, whereas values >.05 may render the model a good fit) and 3 additional fit measures: the comparative fit index (CFI), the incremental fit index (IFI), and the root mean square error of approximation (RMSEA). Values of CFI and IFI above 0.95 were considered adequate, whereas the RMSEA value of 0.05 was below the suggested value of ≤0.06 indicating an acceptable model fit. The CFA was conducted using Amos 21 (IBM SPSS Inc). To measure reliability, we assessed internal consistency and test-retest reliability. The internal consistency of the eHLQ was evaluated using the Cronbach α coefficient (ranges from 0 to 1, with the latter meaning perfect reliability). Test-retest reliability was evaluated using the intraclass correlation coefficient (ICC). Differences between groups were tested by the 2-tailed Students *t* test and ANOVA. Univariable and multivariable linear regression analyses were used to determine factors related to EHL. The results were expressed as linear regression coefficients (β) and their 95% CIs. All tests were 2-tailed. *P*<.05 was considered statistically significant. The IBM SPSS (version 21) package was used for these analyses.

## Results

### Demographic Characteristics of Participants

The Serbian version of the eHLQ questionnaire was completed by 475 PHC users. The mean age of the participants was 51.0 (SD 17.3; range 19-94) years, and most participants were female (328/475, 69.1%). The majority of the participants were married (339/475, 71.4%) and had completed secondary education or higher (409/475, 86.1%). More than half of the participants (278/474, 58.6%) were employees, and 57.1% (266/466) of the sample did not have any longstanding illnesses. The perceived material status was bad in 10.5% (49/466), regular in 50.4% (235/466), and good in 39.1% (182/466). One-half (235/466, 50.4%) of the participants perceived their health as good to excellent, 35.2% (164/466) as regular, and 14.4% (67/466) as bad. A high percentage of participants owned digital devices (431/475, 90.7%), but 28.5% (133/466) did not search for any web-based health information. The participants’ demographics are summarized in Table 1.

Descriptive statistics of eHLQ domain scores are presented in Table 2.

The highest EHL mean scores were obtained for the *understanding of health concepts and language* (mean 2.86, SD 0.32) and *feel safe and in control* (mean 2.89, SD 0.33) domains, while the lowest values were for *using technology to process health information* (mean 2.51, SD 0.33), *digital services that suit individual needs* (mean 2.55, SD 0.27), and *access to digital services that work* (mean 2.57, SD 0.45) domains.

**Table 1:** Participant demographics (N=475).

Variables			Participants, n (%)
**Age (years)**			
	<65	343 (72.2)	
	≥65	132 (27.8)	
**Sex**			
	Male	147 (30.9)	
	Female	328 (69.1)	
**Place of living**			
	City	313 (65.9)	
	Other	162 (34.1)	
**Education**			
	Incomplete secondary education	66 (13.9)	
	Complete secondary education	263 (55.4)	
	Higher education	146 (30.7)	
**Marital status**			
	Married	339 (71.4)	
	Single, separated, or widowed	136 (28.6)	
**Occupation**			
	Employed	278 (58.6)	
	Unemployed	196 (41.4)	
**Perceived material status**			
	Very bad or bad	49 (10.5)	
	Neutral	235 (50.4)	
	Good or very good	182 (39.1)	
**Chronic disease**			
	Yes	200 (42.9)	
	No	266 (57.1)	
**Perceived health status**			
	Very bad or bad	67 (14.4)	
	Neutral	164 (35.2)	
	Good or very good	235 (50.4)	
**Digital device**			
	Yes	431 (90.7)	
	No	44 (9.3)	
**Searching for health information on the internet**			
	Never	133 (28.5)	
	Rarely	271 (58.2)	
	Frequently	62 (13.3)	

**Table 2:** eHLQ^a^ domain scores, internal consistency, and test-retest reliability.

Domains	Score, mean (SD)	Cronbach α	ICC^b^ (95% CI)
1. *Using technology* to process health information	2.51 (0.33)	0.90	0.63 (0.22-0.82)
2. Understanding of *health concepts* and language	2.86 (0.32)	0.81	0.79 (0.57-0.90)
3. Ability to actively *engage* with digital services	2.71 (0.35)	0.90	0.82 (0.62-0.91)
4. *Feel safe* and in control	2.89 (0.33)	0.82	0.81 (0.61-0.91)
5. *Motivated* to engage with digital services	2.59 (0.41)	0.85	0.65 (0.26-0.83)
6. *Access* to digital services that work	2.57 (0.45)	0.83	0.73 (0.43-0.87)
7. Digital services that *suit* individual *needs*	2.55 (0.27)	0.89	0.81 (0.60-0.91)

^a^eHLQ: eHealth Literacy Questionnaire.

^b^ICC: intraclass correlation coefficient.

### Psychometric Properties

**Figure 1:**
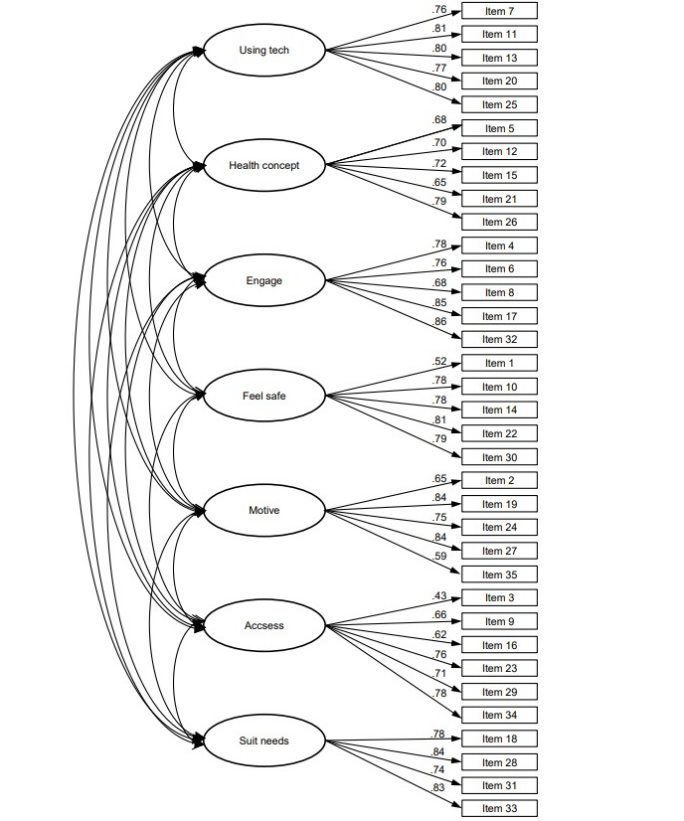
Standardized factor loadings of the 7-factor structure of the eHLQ. eHLQ: eHealth Literacy Questionnaire.

The 7-factor structure of the eHLQ has been validated with maximum likelihood confirmatory analysis, and the results demonstrated a good fit of the data to the hypothesized 7-factor model. The *χ*^2^ test rejected the 7D model (*χ^2^*=1001.9, *P*<.001), as we expected, due to the large sample size. Values for IFI (0.96) and CFI (0.95) were above the cutoff of ≥0.95. The RMSEA value of 0.05 was below the suggested value of ≤0.06. All standardized factor loadings were statistically significant (all *P*<.05) and ranged from 0.43 to 0.86 (see Figure 1).

Analysis of the internal consistency of the Serbian version of the eHLQ showed that the Cronbach α of the entire scale (items 1-35) was 0.95, indicating excellent scale reliability. The α coefficients of the 7 domains were estimated to be 0.90 for domain 1, estimated to be 0.81 for domain 2, estimated to be 0.90 for domain 3, estimated to be 0.82 for domain 4, estimated to be 0.85 for domain 5, estimated to be 0.83 for domain 6, and estimated to be 0.89 for domain 7. For the test-retest, 30 participants completed the retest, and the ICC ranged from 0.63 to 0.82, indicating moderate to good test-retest reliability. Test-retest reliability showed moderate agreement for 2 domains (ICC 0.63 and 0.65) and good agreement for 5 domains (ICC from 0.73 to 0.82; Table 2).

Statistically significant differences (all *P*<.05) for all 7 eHLQ scores were observed for age, education, perceived material status, perceived health status, searching for health information on the internet, and occupation (except domain 4). Participants aged younger than 65 years scored higher in every eHLQ domain when compared with those aged older than 65 years. Female participants had higher scores than male participants in *using technology to process health information* and the *ability to actively engage with digital service* domains. No significant difference was obtained concerning marital status (all *P*>.05). Participants with completed secondary education or higher showed a higher level of EHL compared to those with incomplete secondary education. Employed participants scored higher in every eHLQ domain when compared with the unemployed, except for domain 4. Participants with better-perceived material and health status showed a higher level of EHL (see Table 3).

In multivariable regression models, searching for health information on the internet and aged younger than 65 years were associated with higher values of all domain scores except the domain *feel safe and in control* for variable age (Table 4). Secondary education or higher was positively associated with the domains of *understanding of health concepts and language* and *the ability to actively engage with digital services.* Domains of *ability to actively engage with digital services,* *motivation to engage with digital services, access to digital services that work*, and *digital services that suit individual needs* were all negatively affected by chronic disease. Having a material status that was considered to be good or very good was associated with higher values of domains of *understanding of health concepts and language, feeling safe and in control, access to digital services that work,* and *digital services that suit individual needs.* Place of living and marital status were found to be negatively correlated to the *feel safe and in control* domain.

**Table 3:** Associations among sociodemographic factors and eHLQ^a^ domain scores.

Variables			Using tech	Health concepts	Engage		Feel safe		Motive		Access		Suit needs
**Age (years), mean (SD)**													
	<65	2.66 (0.58)		2.95 (0.44)	2.96 (0.52)	2.93 (0.51)		2.70 (0.56)		2.70 (0.51)		2.71 (0.57)	
	≥65	2.12 (0.61)		2.61 (0.44)	2.08 (0.57)	2.78 (0.49)		2.31 (0.50)		2.24 (0.46)		2.11 (0.50)	
	*P* value (*t* test)	*<.001^b^*		*<.001*	*<.001*	*.003*		*<.001*		*<.001*		*<.001*	
**Sex, mean (SD)**													
	Male	2.42 (0.64)		2.82 (0.43)	2.55 (0.67)	2.88 (0.55)		2.54 (0.58)		2.50 (0.54)		2.50 (0.61)	
	Female	2.55 (0.63)		2.88 (0.48)	2.79 (0.65)	2.89 (0.48)		2.61 (0.57)		2.60 (0.53)		2.58 (0.61)	
	*P* value (*t* test)	*.04*		.21	*<.001*	.79		.27		.07		.10	
**Place of living, mean (SD)**													
	City	2.55 (0.63)		2.88 (0.45)	2.77 (0.65)	2.85 (0.49)		2.58 (0.57)		2.56 (0.55)		2.58 (0.62)	
	Other	2.43 (0.65)		2.81 (0.48)	2.60 (0.68)	2.96 (0.53)		2.59 (0.57)		2.59 (0.62)		2.49 (0.60)	
	*P* value (*t* test)	.051		.12	*.007*	*.03*		.85		.52		.13	
**Education, mean (SD)**													
	Incomplete secondary education	2.45 (0.68)		2.60 (0.53)	2.22 (0.71)	2.77 (0.47)		2.38 (0.55)		2.37 (0.55)		2.22 (0.69)	
	Complete secondary education	2.55 (0.64)		2.89 (0.46)	2.71 (0.64)	2.94 (0.52)		2.64 (0.59)		2.61 (0.54)		2.59 (0.60)	
	Higher education	2.56 (0.57)		2.91 (0.40)	2.93 (0.56)	2.85 (0.49)		2.60 (0.53)		2.59 (0.50)		2.62 (0.55)	
	*P* value (ANOVA)	*.001*		*<.001*	*<.001*	*.03*		*.005*		*.004*		*<.001*	
**Marital status, mean (SD)**													
	Married	2.53 (0.61)		2.85 (0.47)	2.74 (0.64)	2.86 (0.51)		2.58 (0.57)		2.57 (0.53)		2.54 (0.61)	
	Single, separated, or widowed	2.47 (0.69)		2.87 (0.45)	2.66 (0.73)	2.96 (0.49)		2.61 (0.59)		2.58 (0.56)		2.55 (0.64)	
	*P* value (*t* test)	.36		.67	.24	.06		.61		.76		.82	
**Occupation** **, mean (SD)**													
	Employed	2.64 (0.56)		2.94 (0.44)	2.97 (0.49)	2.92 (0.52)		2.68 (0.56)		2.70 (0.50)		2.72 (0.55)	
	Unemployed	2.33 (0.69)		2.74 (0.47)	2.35 (0.72)	2.85 (0.49)		2.46 (0.57)		2.40 (0.53)		2.31 (0.63)	
	*P* value (*t* test)	*<.001*		*<.001*	*<.001*	.12		*<.001*		*<.001*		*<.001*	
**Perceived material status, mean (SD)**													
	Very bad or bad	2.20 (0.62)		2.53 (0.49)	2.22 (0.59)	2.59 (0.57)		2.34 (0.60)		2.23 (0.48)		2.15 (0.55)	
	Neutral	2.44 (0.63)		2.82 (0.45)	2.68 (0.66)	2.88 (0.47)		2.51 (0.57)		2.54 (0.54)		2.50 (0.63)	
	Good or very good	2.65 (0.62)		2.30 (0.43)	2.89 (0.64)	2.98 (0.52)		2.73 (0.54)		2.69 (0.52)		2.69 (0.57)	
	*P* value (ANOVA)	*<.001*		*<.001*	*<.001*	*<.001*		*<.001*		*<.001*		*<.001*	
**Perceived health status, mean (SD)**													
	Very bad or bad	2.31 (0.71)		2.70 (0.52)	2.26 (0.69)	2.81 (0.51)		2.43 (0.57)		2.36 (0.51)		2.31 (0.58)	
	Neutral	2.40 (0.64)		2.76 (0.50)	2.57 (0.64)	2.83 (0.52)		2.49 (0.61)		2.46 (0.55)		2.38 (0.61)	
	Good or very good	2.63 (0.59)		2.98 (0.39)	2.94 (0.59)	2.95 (0.49)		2.70 (0.53)		2.70 (0.50)		2.72 (0.59)	
	*P* value (ANOVA)	*<.001*		*<.001*	*<.001*	*.02*		*<.001*		*<.001*		*<.001*	
**Searching for health information on the internet, mean (SD)**													
	Never	1.96 (0.56)		2.60 (0.48)	2.14 (0.67)	2.78 (0.53)		2.23 (0.55)		2.28 (0.53)		2.14 (0.62)	
	Rarely	2.67 (0.52)		2.96 (0.40)	2.94 (0.51)	2.93 (0.50)		2.70 (0.53)		2.67 (0.48)		2.69 (0.54)	
	Frequently	2.92 (0.54)		2.95 (0.51)	2.96 (0.54)	2.96 (0.50)		2.82 (0.49)		2.72 (0.54)		2.76 (0.57)	
	*P* value (ANOVA)	*<.001*		*<.001*	*<.001*	*.008*		*<.001*		*<.001*		*<.001*	

^a^eHLQ: eHealth Literacy Questionnaire.

^b^Significant *P* values are italicized.

**Table 4:** Regression models of variables associated with eHLQ^a^ domain scores.

eHLQ domains			β (95% CI)		*P* value
**Using technology**					
	Searching for health information on the internet	0.46 (0.37 to 0.54)		*<.001^b^*	
	Age (years)	0.29 (0.17 to 0.40)		*<.001*	
**Health concepts**					
	Age (years)	0.21 (0.11 to 0.30)		*<.001*	
	Searching for health information on the internet	0.13 (0.06 to 0.20)		*<.001*	
	Material status	0.19 (0.06 to 0.33)		*.006*	
	Education	0.15 (0.02 to 0.27)		*.02*	
**Engage**					
	Age (years)	0.61 (0.49 to 0.73)		*<.001*	
	Searching for health information on the internet	0.29 (0.21 to 0.37)		*<.001*	
	Chronic disease	–0.17 (–0.27 to –0.06)		*.001*	
	Education	0.20 (0.06 to 0.35)		*.005*	
**Feel safe**					
	Material status	0.33 (0.18 to 0.48)		*<.001*	
	Place of living	–0.15 (–0.24 to –0.05)		*.003*	
	Searching for health information on the internet	0.10 (0.03 to 0.18)		*.005*	
	Marital status	–0.13 (–0.23 to –0.03)		*.01*	
**Motive**					
	Searching for health information on the internet	0.28 (0.20 to 0.36)		*<.001*	
	Chronic disease	–0.14 (–0.25 to –0.03)		*.01*	
	Age (years)	0.16 (0.04 to 0.29)		*.01*	
**Access**					
	Age (years)	0.28 (0.16 to 0.40)		*<.001*	
	Searching for health information on the internet	0.16 (0.08 to 0.24)		*<.001*	
	Chronic disease	–0.11 (–0.21 to –0.01)		*.04*	
	Material status	0.16 (0.01 to 0.31)		*.04*	
**Suit needs**					
	Age (years)	0.39 (0.26 to 0.52)		*<.001*	
	Searching for health information on the internet	0.23 (0.15 to 0.32)		*<.001*	
	Chronic disease	–0.13 (–0.24 to –0.02)		*.02*	
	Material status	0.17 (0.003 to 0.34)		*.046*	

^a^eHLQ: eHealth Literacy Questionnaire.

^b^Significant *P* values are italicized.

## Discussion

### Principal Findings

One of the primary goals of the digitalization program in the Republic of Serbia’s health care system is to establish and support the development of a health system that places the patient at the center (also known as a “patient-centered approach”). More than 80 patient associations, organizations, and associations were contacted during the preparation of this document; they acknowledged the benefits of digitization, but they also identified several needs and difficult aspects, such as the low levels of the EHL of their members, as well as all patients in Serbia [26].

In this study, we used a well-defined translation and cultural adaptation process to reproduce the original instrument’s concepts and meanings. We evaluated the psychometric properties of the Serbian version of the eHLQ in the population of PHC users and explored factors associated with EHL. Our data from a sample of PHC users from urban and rural municipalities demonstrated that the Serbian eHLQ has strong psychometric properties and is in line with the psychometric outcomes of the versions in English and other languages [1,27,33,34].

Based on previous recommendations for examining the validity of a translated instrument, this study used qualitative and quantitative approaches. During the first phase, we adopted the TIP, which includes a multistep translation and review process and detailed item intent descriptions [30,31]. The results of the cognitive interviews and several review board meetings resulted in our revising a few words that were considered problematic in a Serbian context; however, most items on the Serbian eHLQ were understood as intended, and their equivalence to the original and translated versions was maintained [28].

The overall findings of this study provide evidence for the good validity and reliability of the Serbian eHLQ. CFA validated the 7-factor structure of the questionnaire. According to the preestablished CFI, IFI, and RMSEA thresholds, the original 7D model can be considered acceptable. Only the *χ*^2^ test revealed a bad fit for the 7D model analyzed due to the large sample size. All standardized factor loadings were statistically significant and ranged from 0.43 to 0.86. These findings are per previous validation studies conducted in other populations that confirmed the 7-factor structure of the questionnaire [1,27,28,34].

Analysis of the internal consistency of the Serbian version of the eHLQ showed that the Cronbach α of the entire scale was 0.95, indicating excellent scale reliability. All scales demonstrated good internal consistency, with a Cronbach α of >0.80, and ICC ranged from 0.63 to 0.82, indicating moderate to good test-retest reliability. The lowest value was for domain 2*, understanding of health concepts and language* [27,28,33], which was consistent with Danish, Australian, Taiwanese, and Swedish eHLQ validation studies.

The majority of the participants in our study were married (339/475, 71.4%) and had completed secondary education or higher (409/475, 86.1%). More than half of the participants were employed (278/474, 58.6%) and did not have any longstanding illnesses (266/466, 57.1%). Every 10th PHC user (49/466, 10.5%) in our sample had a bad perceived material status, while half (235/466, 50.4%) of the participants considered their health to be good to excellent. A high percentage (431/475, 90.7%) of participants owned digital devices, but more than a quarter (133/466, 28.5%) of the sample did not search for any web-based health information, showing that patients with limited use of eHealth were well represented in this study’s sample.

The highest EHL mean scores were obtained for domain 2, *understanding of health concepts and language,* and domain 4, *feel safe and in control*, while the lowest values were for domain 1, *using technology to process health information;* domain 7, *digital services that suit individual needs;* and domain 6, *access to digital services that work*. The lowest scores were obtained for domains that depend mostly on interaction with DH services and the accessibility of technology. This finding concurred with the results of a study by García-García et al [8].

Previous studies have already shown that EHL significantly depends on sociodemographic factors [7,8,17]. In our study, statistically significant differences for all 7 eHLQ scores were observed for age, education, perceived material status, perceived health status, searching for health information on the internet, and occupation (except domain 4, *feel safe and in control*). Female participants had higher scores than male participants in domain 1, *using technology to process health information*, and domain 3, *ability to actively engage with digital services.* Literature data on sex influencing EHL was inconclusive because some studies have positioned female sex as a protective factor while other studies have reported higher scores in male participants [8]. Based on previous research on EHL in Serbia, the female sex has been consistently associated with better EHL across age-specific populations, and female individuals tend to use more primary and specialized care compared to men [35].

Participants aged younger than 65 years scored higher in every eHLQ domain when compared with those aged older than 65 years. Age-specific results are similar in other studies, which have stated that older age is associated with decreased adoption and use of health care technologies and holds the most prejudice against them [1,8,36].

Participants with completed secondary education or higher showed a higher level of EHL compared to those with incomplete secondary education, as in other studies [1,7,33,36]. The population with completed secondary education or higher in our study had statistically significantly higher eHLQ scores, except for domain 4, *feel safe and in control*; domain 5, *motivated to engage with digital services*; and domain 6, *access to digital services that work*. People with lower education used eHealth less often [1,34,36]. However, having more education did not mean that the patients felt safer or had better access to eHealth. These results are consistent with some literature data, but the higher score could also be a result of a difference in interpretation between these 2 groups [1,8,33]. The Serbian version of the eHLQ could be a promising tool for understanding digital access at different educational levels, as in other contexts [34].

No significant difference was obtained concerning marital status despite the study of García-García et al [8], where those who were single, separated, or widowed scored significantly lower for domain 1, *using technology to process health information*; domain 4, *feel safe and in control*; domain 5, *motivated to engage with digital services*; and domain 6, *access to digital services that work*, indicating that people who are “alone” might face challenges in these areas.

Employed participants scored higher in every eHLQ domain when compared with the unemployed, except for domain 4. The majority of jobs in contemporary society require good digital skills [34].

In our study, searching for health information on the internet and those aged younger than 65 years were associated with higher values of all domain scores except domain 4, *feel safe and in control*, for variable age. Participants with better-perceived material and health status were found to have a positive association with better EHL, except for domain 4, *feel safe and in control*. Domain 3*, ability to actively engage with digital services*; domain 5*, motivated to engage with digital services*; domain 6, *access to digital services that work*; and domain 7, *digital services that suit individual needs* were all negatively affected by chronic disease. In line with the data from the literature, health status is one of the crucial determinants of health care technology adoption [8].

Although previous instruments, such as the Serbian version of the eHealth Literacy Scale, have focused on individuals’ competencies, the eHLQ has the added perspective of interaction between the individual and the eHealth systems in Serbia [27,35].

### Limitations

Considering that this was a cross-sectional study, it is impossible to determine causality. Only associations can be interpreted from this data. Collected data may also be subject to errors due to subjective reporting or the selective memory of respondents. This study included only PHC patients from the Mačva district of Western Serbia. Future testing of the Serbian eHLQ in different contexts in Serbia may strengthen the validity of the instrument.

### Conclusions

This study provided evidence for the appropriate metric properties of the Serbian version of eHLQ. Searching for health information on the internet and age were factors influencing almost all scale domains. This study demonstrates that the Serbian version of the eHLQ can be a useful instrument in measuring EHL and in planning eHealth interventions at the population and individual levels. It is a useful tool for understanding the socioeconomic determinants of digital access inequity. The Serbian eHLQ can represent a basis for further research, and its results could establish complex connections with the way users of health system services, health workers, and digital technologies interact, which will help policymakers evaluate and implement new eHealth interventions.

## Abbreviations

CFA: confirmatory factor analysis
CFI: comparative fit index
DH: digital health
EHL: eHealth literacy
eHLQ: eHealth Literacy Questionnaire
ICC: intraclass correlation coefficient
IFI: incremental fit index
PHC: primary health care
RMSEA: root mean square error of approximation
TIP: Translation Integrity Procedure

## Data Availability

About the Questionnaire Licence Agreement, Swinburne University of Technology is the owner of the license for use of the Serbian version of the eHealth Literacy Questionnaire (eHLQ), and if you would like to access this tool, please contact Ms Kerrie Paulger at kpaulger@swin.edu.au. All other data sets generated or analyzed during this study are not publicly available due to the Institute of Public Health of Sabac’s policy but are available from the corresponding author on reasonable request.

## References

[1] Poot CC, Meijer E, Fokkema M, Chavannes NH, Osborne RH, Kayser L (2023). Translation, cultural adaptation and validity assessment of the Dutch version of the eHealth Literacy Questionnaire: a mixed-method approach. BMC Public Health.

[2] (2016). From Innovation to Implementation: eHealth in the WHO European Region.

[3] Prestidge M, Cafagna G, Dalton CW, de Oliviera T, Feliciano JC, Feng SC, Goswami KP (2023). Digital in health: unlocking the value for everyone. The World Bank.

[4] Davaris MT, Bunzli S, Trieu J, Dowsey MM, Choong PF (2022). The role of digital health interventions to improve health literacy in surgical patients: a narrative review in arthroplasty. ANZ J Surg.

[5] Busse TS, Nitsche J, Kernebeck S, Jux C, Weitz J, Ehlers JP, Bork U (2022). Approaches to improvement of digital health literacy (eHL) in the context of person-centered care. Int J Environ Res Public Health.

[6] Conard S (2019). Best practices in digital health literacy. Int J Cardiol.

[7] Lwin MO, Panchapakesan C, Sheldenkar A, Calvert GA, Lim LKS, Lu J (2020). Determinants of eHealth literacy among adults in China. J Health Commun.

[8] García-García D, Bazán MJA, Pérez-Rivas FJ (2022). Factors influencing eHealth literacy among Spanish primary healthcare users: cross-sectional study. Int J Environ Res Public Health.

[9] Campanozzi LL, Gibelli F, Bailo P, Nittari G, Sirignano A, Ricci G (2023). The role of digital literacy in achieving health equity in the third millennium society: a literature review. Front Public Health.

[10] Monkman H, Kushniruk AW (2015). eHealth literacy issues, constructs, models, and methods for health information technology design and evaluation. Knowl Manag E-Learn.

[11] Eysenbach G (2002). Infodemiology: the epidemiology of (mis)information. Am J Med.

[12] Eysenbach G (1999). Rating information on the internet can empower users to make informed decisions. BMJ.

[13] Eysenbach G, Jadad AR (2001). Evidence-based patient choice and consumer health informatics in the internet age. J Med Internet Res.

[14] Melhem SJ, Nabhani-Gebara S, Kayyali R (2023). Digital trends, digital literacy, and e-health engagement predictors of breast and colorectal cancer survivors: a population-based cross-sectional survey. Int J Environ Res Public Health.

[15] Moorhead SA, Hazlett DE, Harrison L, Carroll JK, Irwin A, Hoving C (2013). A new dimension of health care: systematic review of the uses, benefits, and limitations of social media for health communication. J Med Internet Res.

[16] Blandford A, Wesson J, Amalberti R, AlHazme R, Allwihan R (2020). Opportunities and challenges for telehealth within, and beyond, a pandemic. Lancet Glob Health.

[17] Estrela M, Semedo G, Roque F, Ferreira PL, Herdeiro MT (2023). Sociodemographic determinants of digital health literacy: a systematic review and meta-analysis. Int J Med Inform.

[18] Mee P, Gussy M, Huntley P Digital exclusion as a barrier to accessing healthcare: a summary composite indicator and online tool to explore and quantify local differences in levels of exclusion. medRxiv.

[19] Sundell E, Wångdahl J, Grauman Å (2022). Health literacy and digital health information-seeking behavior—a cross-sectional study among highly educated Swedes. BMC Public Health.

[20] Dong Q, Liu T, Liu R, Yang H, Liu C (2023). Effectiveness of digital health literacy interventions in older adults: single-arm meta-analysis. J Med Internet Res.

[21] Kim S, Chow BC, Park S, Liu H (2023). The usage of digital health technology among older adults in Hong Kong and the role of technology readiness and eHealth literacy: path analysis. J Med Internet Res.

[22] Kouri A, Gupta S, Straus SE, Sale JEM (2023). Exploring the perspectives and experiences of older adults with asthma and chronic obstructive pulmonary disease toward mobile health: qualitative study. J Med Internet Res.

[23] Abel T, McQueen D (2020). Critical health literacy and the COVID-19 crisis. Health Promot Int.

[24] Cui GH, Li SJ, Yin YT, Chen LJ, Li JQ, Liang FY, Liu XY, Chen L (2021). The relationship among social capital, eHealth literacy and health behaviours in Chinese elderly people: a cross-sectional study. BMC Public Health.

[25] van der Vaart R, Drossaert C (2017). Development of the digital health literacy instrument: measuring a broad spectrum of health 1.0 and health 2.0 skills. J Med Internet Res.

[26] (2022). Digitalization program in the healthcare system of the Republic of Serbia for the period 2022-2026. Government of Republic of Serbia.

[27] Sjöström AE, Hajdarevic S, Hörnsten Å, Kristjánsdóttir Ó, Castor C, Isaksson U (2023). The Swedish version of the eHealth Literacy Questionnaire: translation, cultural adaptation, and validation study. J Med Internet Res.

[28] Kayser L, Karnoe A, Furstrand D, Batterham R, Christensen KB, Elsworth G, Osborne RH (2018). A multidimensional tool based on the eHealth literacy framework: development and initial validity testing of the eHealth Literacy Questionnaire (eHLQ). J Med Internet Res.

[29] Rademakers J, Waverijn G, Rijken M, Osborne R, Heijmans M (2020). Towards a comprehensive, person-centred assessment of health literacy: translation, cultural adaptation and psychometric test of the Dutch Health Literacy Questionnaire. BMC Public Health.

[30] Hawkins M, Cheng C, Elsworth GR, Osborne RH (2020). Translation method is validity evidence for construct equivalence: analysis of secondary data routinely collected during translations of the Health Literacy Questionnaire (HLQ). BMC Med Res Methodol.

[31] Beatty PC, Willis GB (2007). Research synthesis: the practice of cognitive interviewing. Public Opin Q.

[32] World Medical Association (2013). World Medical Association declaration of Helsinki: ethical principles for medical research involving human subjects. JAMA.

[33] Cheng C, Elsworth GR, Osborne RH (2022). Validity evidence of the eHealth Literacy Questionnaire (eHLQ) part 2: mixed methods approach to evaluate test content, response process, and internal structure in the Australian Community Health setting. J Med Internet Res.

[34] Chen YC, Cheng C, Osborne RH, Kayser L, Liu CY, Chang LC (2022). Validity testing and cultural adaptation of the eHealth Literacy Questionnaire (eHLQ) among people with chronic diseases in Taiwan: mixed methods study. J Med Internet Res.

[35] Gazibara T, Cakic J, Cakic M, Pekmezovic T, Grgurevic A (2019). eHealth and adolescents in Serbia: psychometric properties of eHeals questionnaire and contributing factors to better online health literacy. Health Promot Int.

[36] Xesfingi S, Vozikis A (2016). eHealth literacy: in the quest of the contributing factors. Interact J Med Res.

